# Increasing Clinical Severity during a Dengue Virus Type 3 Cuban Epidemic: Deep Sequencing of Evolving Viral Populations

**DOI:** 10.1128/JVI.02647-15

**Published:** 2016-04-14

**Authors:** Rosmari Rodriguez-Roche, Hervé Blanc, Antonio V. Bordería, Gisell Díaz, Rasmus Henningsson, Daniel Gonzalez, Emidalys Santana, Mayling Alvarez, Osvaldo Castro, Magnus Fontes, Marco Vignuzzi, Maria G. Guzman

**Affiliations:** a Virology Department, Pedro Kouri Institute of Tropical Medicine, PAHO/WHO Collaborating Center for the Study of Dengue and Its Vector, Havana, Cuba; b Institut Pasteur, Viral Populations and Pathogenesis Unit, CNRS UMR 3569, Paris, France; c Institut Pasteur, International Group for Data Analysis, Paris, France; d Lund University, Centre for Mathematical Sciences, Lund, Sweden; e Department of Medicine, Pedro Kouri Institute of Tropical Medicine, Havana, Cuba

## Abstract

During the dengue virus type 3 (DENV-3) epidemic that occurred in Havana in 2001 to 2002, severe disease was associated with the infection sequence DENV-1 followed by DENV-3 (DENV-1/DENV-3), while the sequence DENV-2/DENV-3 was associated with mild/asymptomatic infections. To determine the role of the virus in the increasing severity demonstrated during the epidemic, serum samples collected at different time points were studied. A total of 22 full-length sequences were obtained using a deep-sequencing approach. Bayesian phylogenetic analysis of consensus sequences revealed that two DENV-3 lineages were circulating in Havana at that time, both grouped within genotype III. The predominant lineage is closely related to Peruvian and Ecuadorian strains, while the minor lineage is related to Venezuelan strains. According to consensus sequences, relatively few nonsynonymous mutations were observed; only one was fixed during the epidemic at position 4380 in the NS2B gene. Intrahost genetic analysis indicated that a significant minor population was selected and became predominant toward the end of the epidemic. In conclusion, greater variability was detected during the epidemic's progression in terms of significant minority variants, particularly in the nonstructural genes. An increasing trend of genetic diversity toward the end of the epidemic was observed only for synonymous variant allele rates, with higher variability in secondary cases. Remarkably, significant intrahost genetic variation was demonstrated within the same patient during the course of secondary infection with DENV-1/DENV-3, including changes in the structural proteins premembrane (PrM) and envelope (E). Therefore, the dynamic of evolving viral populations in the context of heterotypic antibodies could be related to the increasing clinical severity observed during the epidemic.

**IMPORTANCE** Based on the evidence that DENV fitness is context dependent, our research has focused on the study of viral factors associated with intraepidemic increasing severity in a unique epidemiological setting. Here, we investigated the intrahost genetic diversity in acute human samples collected at different time points during the DENV-3 epidemic that occurred in Cuba in 2001 to 2002 using a deep-sequencing approach. We concluded that greater variability in significant minor populations occurred as the epidemic progressed, particularly in the nonstructural genes, with higher variability observed in secondary infection cases. Remarkably, for the first time significant intrahost genetic variation was demonstrated within the same patient during the course of secondary infection with DENV-1/DENV-3, including changes in structural proteins. These findings indicate that high-resolution approaches are needed to unravel molecular mechanisms involved in dengue pathogenesis.

## INTRODUCTION

Dengue viruses (DENVs) cause the most important arthropod-borne viral disease in humans, with latest estimates of 390 million dengue infections per year, of which 96 million manifest some level of disease severity (1). This figure is more than three times the dengue burden estimate of the World Health Organization (2). Latin America has progressively evolved a region with low dengue endemicity to a region of hyperendemicity, with local transmission of the four dengue virus serotypes (DENV 1 to 4) in practically all countries (3). DENVs are assigned to the genus Flavivirus in the family Flaviviridae. The genomes of flaviviruses comprise a single-stranded RNA molecule encoding three structural proteins, the capsid (C), premembrane/membrane (PrM/M), and envelope (E), and seven nonstructural (NS) proteins, NS1, NS2A, NS2B, NS3, NS4A, NS4B, and NS5 (4). Infection with any DENV serotype may present as an asymptomatic infection or as a symptomatic infection ranging from mild to severe illness: dengue fever (DF) and dengue hemorrhagic fever/dengue shock syndrome (DHF/DSS) (5) or dengue and severe dengue, according to the new dengue case classification (2).

The etiology of DHF/DSS has been the subject of research for over 40 years (6, 7). In Cuba, secondary infection has been implicated as the most relevant risk factor to developing severe disease, combined with the introduction of DENV strains of Asian origin. Likewise, other risk factors, including age, genetic background, and chronic diseases, like bronchial asthma, sickle cell anemia, and diabetes, have also been implicated in dengue severity (8, 9). Notably, during two severe epidemics (1981 and 1997) characterized by the circulation of only one serotype (DENV-2), 4 and 20 years, respectively, after the massive DENV-1 epidemic, a marked month-to-month increase in clinical severity was observed in secondary infection cases (8, 10, 11).

In June 2001, the nationwide dengue case surveillance system identified a DENV-3 outbreak in Havana City, which eventually involved 12,889 confirmed cases, including 78 DHF/DSS and three fatalities (12). Here, we report that significant monthly increases in the proportion of DHF/DSS cases also occurred during this epidemic, 24 years after the DENV-1 epidemic and 20 years after one caused by DENV-2. Since the epidemic was circumscribed to Havana City, which has an admixed population with homogenous ethnic composition, and since all severe cases were in adults, the hypothesis related to the outbreak moving into different regions with different population demographics to explain the increasing severity was refuted. Likewise, host factors did not appear to explain this increase because it is not logical to assume that the most susceptible individuals would all be infected toward the end of the epidemic.

However, the theory that cross-immunity could play a significant role in shaping viral population diversity, selecting for more fit viruses toward the end of the epidemics that produce severe dengue, is plausible. Previous studies in this particular context have suggested that the phenomenon of increasing clinical severity with the epidemic's progression could be related to temporary changes that occur in the virus causing the epidemic (10, 13, 14). In this regard, little is known about the extent of intrahost genetic diversity of DENVs in relation to immune status and their implications for dengue pathogenesis and disease severity. Initial studies using cloning techniques demonstrated higher levels of DENV-3 intrahost genetic diversity in patients than in mosquitoes (15). In addition, the extent and pattern of DENV-1 diversity during acute infection were related to disease outcome (16), while no relationship was found for the same serotype in a different scenario (17). Later on, studies using deep-sequencing approaches, although in indifferent epidemiological contexts, have shown low genetic diversity in humans (18, 19). Most recent papers on dengue intrahost diversity have been focused on viral population variations during human/mosquito host switching (20–22).

In the present study, we explored intrahost DENV-3 genetic diversity in serum samples collected at different time points during the 2001-2002 Cuban epidemic, using whole-genome amplification and next-generation sequencing to determine the role of the virus in increasing disease severity. Our results demonstrate that changes in the viral population occurring with the epidemic's progression could have an impact on viral fitness.

## MATERIALS AND METHODS

### Epidemic characterization in terms of dengue severity.

The proportion of DHF/DSS per dengue case was compared every four epidemiological weeks as an expression of increasing clinical severity, based on confirmed dengue case figures that were notified during the epidemic period through the laboratory surveillance system (12). Analysis for a linear trend in proportion was done by a chi-square test using Epinfo, version 3.2. Odds ratios (OR) were calculated taking the weeks 33 to 36 as a reference because no DHF/DSS cases were observed in preceding weeks.

### Samples.

Twenty-two DENV-3 acute positive serum samples corresponding to 21 patients collected at different time points during the 2001-2002 Cuban epidemic were utilized for molecular characterization (Table 1). Two samples (Cuba_553_2001 and Cuba_558_2001) correspond to the same patient. All samples had been processed during the epidemic period for viral isolation on C6/36 HT cells and identification by indirect immune fluorescence with monoclonal antibodies (23) and then stored at −80°C in the strain bank of the National Reference Laboratory of Virology at Pedro Kouri Institute of Tropical Medicine. Informed consent was obtained from all patients at the moment of sample collection. All cases were classified clinically at that time as DF or DHF/DSS according to the Guidelines for Control and Prevention of Dengue and Dengue Hemorrhagic Fever in the Americas (24). In addition, the most recent clinical classification (2) was obtained after analysis of data available in the clinical records of the patients. Ten patients included in the study, classified as DF because they did not fulfill the strict WHO criteria to classify them as DHF according to the 1997 guidelines, presented warning signs and required hospitalization. Indeed, they were at risk of severe dengue. However, early hospitalization policies combined with proper clinical management prevented complications. Patients without warning signs treated at home were visited daily by the family doctor to accurately define the final disease outcome. The Institutional Ethical Review Committee of the Pedro Kouri Institute of Tropical Medicine approved the present study (IRB number CEI-IPK-13-11).

**TABLE 1:** Data of DENV-3 samples collected during the 2001-2002 epidemic

Sample no.	Sample code	Date of sample collection (day-mo-yr)	No. of days of illness	Municipality	Clinical classification according to:		Type of infection
					WHO^*a*^	WHO/TDR^*b*^	
1	Cuba_15_01	5-7-01	1	Playa	DF	Dengue, −WS	Secondary
2	Cuba_26_01	15-7-01	2	La Lisa	DF	Dengue, −WS	Secondary
3	Cuba_73_01	22-7-01	2	Playa	DF	Dengue, −WS	Secondary
4	Cuba_118_01	3-8-01	2	Arroyo Naranjo	DF	Dengue, −WS	Primary
5	Cuba_167_01	15-8-01	2	Arroyo Naranjo	DF	Dengue, −WS	Primary
6	Cuba_463_01	23-9-01	2	Playa	DHF/DSS^*c*^	Severe dengue	Secondary
7	Cuba_492_01	28-9-01	2	Playa	DF^*c*^	Dengue, +WS	Secondary
8	Cuba_504_01	1-10-01	3	Playa	DF^*c*^	Dengue, +WS	Secondary
9	Cuba_513_01	2-10-01	3	Playa	DF^*c*^	Dengue, +WS	Primary
10	Cuba_523_01	5-10-01	2	Playa	DF^*c*^	Dengue, +WS	Primary
11	Cuba_546_01	15-10-01	2	Marianao	DF^*c*^	Dengue, +WS	Secondary
12	Cuba_547_01	15-10-01	2	Arroyo Naranjo	DF^*c*^	Dengue, +WS	Primary
13^*e*^	Cuba_553_01	21-10-01	2	Marianao	DF^*c*^	Dengue, +WS	Secondary
14	Cuba_557_01	22-10-01	2	Marianao	DF^*c*^	Dengue, +WS	Primary
15^*e*^	Cuba_558_01	21-10-01	4	Marianao	DF^*c*^	Dengue, +WS	Secondary
16	Cuba_568_01	8-11-01	2	La Lisa	DF	Dengue, −WS	Secondary
17	Cuba_580_01	22-11-01	4	La Lisa	DHF/DSS^*c*^^,^^*d*^	Severe dengue^*d*^	Secondary
18	Cuba_16_02	6-1-02	3	Habana Vieja	DF	Dengue, −WS	Primary
19	Cuba_11_02	15-1-02	2	Plaza	DF^*c*^	Dengue, +WS	Secondary
20	Cuba_17_02	20-1-02	2	Centro Habana	DF	Dengue, −WS	Secondary
21	Cuba_20_02	20-1-02	2	Centro Habana	DF	Dengue, −WS	Secondary
22	Cuba_21_02	21-1-02	1	Centro Habana	DF	Dengue, −WS	Primary

a See reference 5. DF, Dengue fever; DHF/DSS, dengue hemorrhagic fever/dengue shock syndrome.

b See reference 2. +WS, with warning signs; −WS, without warning signs.

c Required hospitalization.

d Fatal case.

e Samples 13 and 15 represent the same patient.

### IgG detection.

All samples were processed by an enzyme-linked immunosorbent assay (ELISA) inhibition test to determine anti-dengue virus IgG titers. Samples with titers less than 1/20 were considered primary infections, and samples with titers higher than 1/1,280, were considered secondary infections (25).

### Sequence of infection.

Sera from patients in the convalescent phase of the infection were analyzed for neutralizing antibodies to all DENV serotypes using the 50% endpoint plaque reduction neutralization test described by Morens et al. (26), with some modifications (27). According to criteria previously established (28), patients with neutralizing antibody titers of ≥1:30 to only one DENV serotype were considered to have experienced a primary dengue virus infection. Patients with neutralizing antibody titers of ≥1:30 against two or more serotypes were considered to have experienced a second or third infection.

### Full-length viral genome amplification.

Briefly, viral RNA was extracted from 140 μl of serum sample using a QIAamp viral RNA minikit (Qiagen, Germany). cDNA was synthesized using a Transcriptor High Fidelity cDNA Synthesis kit (Roche Applied Science, Germany) using 600 μM hexamer random primer according to the manufacturer's instructions. An aliquot of 3 μl of cDNA was subjected to PCR using an Expand High Fidelity Plus PCR system (Roche Applied Science, Germany) according to the manufacturer's instructions. Two independent DNA libraries (a and b) were constructed using two different sets of primers for each sample. Five pairs of primers were utilized to obtain five overlapping fragments of 2 to 3 kb (fragments F1a to F5a), covering the complete genome of the DENV-3 virus as previously published (29). In addition, a second set of primers was designed (available from the authors on request); these fragments were named F1b to F5b.

### Population diversity determined by deep sequencing.

To estimate the population diversity of variants by deep sequencing, PCR fragments were purified via a QIAQuick PCR purification kit (Qiagen, Germany), and total DNA was quantified by Pico Green fluorescence (Invitrogen, USA). Amplicons were then fragmented using Fragmentase and linked to Illumina multiplex adapters; they were subjected to clustering and sequencing with Illumina cBot and GAIIX technology and analyzed with established deep-sequencing data analysis tools. Illumina technology was selected since it is capable of producing enough sequencing data to enable identification of rare variants present in as low as a 1:1,000 ratio in samples of modest reference size. Improvements in accuracy were made with the ViVAN (Viral Variant ANalysis) pipeline (30), a robust algorithm based on each variant allele's initial rate and read qualities, to differentiate between sequencing errors and actual population variants, facilitating accurate variant assessment for DENV populations. To minimize inaccuracy due to sequencing errors, alleles at frequencies of >0.1% were analyzed, while ultrarare variants were discarded.

During the sequencing assembly process, a closely related reference sequence (DENV-3/EC/BID-V2975/2000) was utilized. This sequence was selected according to the phylogenetic tree constructed using DENV-3 consensus sequences obtained by the Sanger method. Once we were able to assemble the sequence of the first isolate of the epidemic (Cuba_15_2001), this consensus sequence was used as a reference to analyze minor variants that appeared in DENV-3 samples collected at different time points during the Cuban epidemic. A new statistically significant variant was considered unique for a particular sample if it appeared in both independent DNA libraries. Briefly, for each position throughout the viral genome, base identity and quality score were gathered. Each variant was determined to be true using a generalized-likelihood ratio test (used to determine the total number of minority variants), and its allele rate was modified according to its covering read qualities based on maximum-likelihood estimation. Additionally, a confidence interval was calculated for each allele rate. In order to correct for multiple testing, a Benjamini-Hochberg false-discovery rate of 5% was set (31). Different ViVAN output files were utilized for analysis, such as synonymous and nonsynonymous changes for each significantly variable position, organized by gene and position for the whole viral genome, and a battery of metrics, including nucleotide substitution matrix, transition/transversion frequencies, and variant allele rates. The variant allele rate is the frequency of a significant nonreference allele (in this case, a single nucleotide polymorphism [SNP]) present in the sample, whether it occurs across the whole viral genome or in a specific gene. This measurement is a proxy to calculate heterogeneity of a specific sample. In addition, three files with pairwise comparisons supplying the mutations found to be different or common among samples as well as the consensus sequence files were utilized (30).

Intersample cluster analyses were performed by first computing the distance matrix among all samples using root mean square deviation (RMSD) values according to Li et al. (32). Multidimensional scaling (MDS), with Kruskal's stress criterion (33), was performed on the distance matrix to produce a low-dimensional plot of the samples. Dendrograms based on complete linkage hierarchical clustering of the distance matrix were also plotted.

### Consensus sequence analysis.

Consensus full polyprotein nucleotide sequences of each DENV-3 isolate obtained in the present study were aligned using ClustalX (34), together with relevant sequences retrieved from GenBank (available from the authors on request) such that representative sequences from all the known DENV-3 genotypes were present. From the initial data set, identical sequences and known recombinant sequences were removed from the alignments. This produced a total data set of 104 sequences of 10,170 nucleotides in length. Phylogenetic analyses were performed using Bayesian analysis in MrBayes, version 3.1.2 (35), with a minimum of 20 million generations and a burn-in of 10%. Stationarity was assessed at effective sample size (ESS; >400) using Tracer, version 1.4.1 (part of the BEAST package) (36).

### Nucleotide sequence accession numbers.

The consensus nucleotide sequences reported in this study are available in GenBank under accession numbers KT726340 to KT726361.

## RESULTS

### Increasing clinical severity during the epidemic.

The proportions of DHF/DSS cases per dengue case were compared every four epidemiological weeks as an expression of increasing clinical severity. According to epidemiological data the first dengue case was reported by epidemiological week 22 (beginning of June). However, the first DHF/DSS case occurred by epidemiological week 36 (beginning of September). From weeks 45 to 48 the number of confirmed dengue cases had a tendency to decrease while the proportion of DHF/DSS cases increased notably (*P* < 0.05). The last seven DHF/DSS cases appeared during January 2002, with five of them occurring during the first week. The risk of severe dengue increased noticeably from September 2001 to January 2002 by 7.25-fold (Table 2). These findings confirmed that increasing clinical severity occurred toward the end of the epidemic. Notably, the peak of the epidemic occurred in October; however, the first two fatalities were reported at the end of November 2001, and the last one was reported at the beginning of January 2002, for a total of three during the epidemic period.

**TABLE 2:** Increasing clinical severity during the DENV-3 Cuban epidemic, 2001 to 2002

Parameter	Value for the parameter at the indicated period (wk)^*a*^					
	22–32	33–36	37–40	41–44	45–48	49–52
No. of confirmed dengue cases	187	534	1,857	3,185	2,647	2,356
No. of DHF/DSS cases	0	1	6	7	23	32
No. of deaths	0	0	0	2	0	1
Proportion of dengue cases with DHF/DSS (%)^*b*^	0	0.2	0.3	0.2	0.9	1.4
Odds ratio		1	1.72	1.17	4.64	7.25

a Epidemiological weeks span the following months: weeks 22 to 32, June to August 2001; weeks 33 to 36, August to September 2001; weeks 37 to 40, September to October 2001, weeks 41 to 44, October to November 2001; weeks 45 to 48, November to December 2001; weeks 49 to 52, December 2001 to January 2002.

b Values based on a chi-square test for the linear trend from epidemiological weeks 33 to 36 and weeks 49 to 52 (*P* < 0.05).

### Phylogenetic analysis.

**FIG 1:**
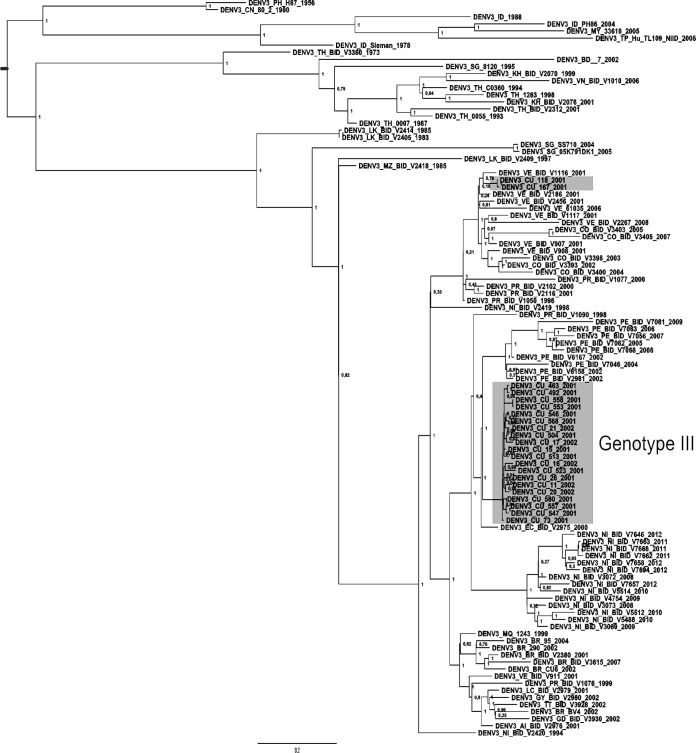
Bayesian phylogeny of the DENV-3 polyprotein data set, including Cuban isolates from the 2001-2002 epidemic highlighted in gray. All horizontal branch lengths are drawn to scale. Bar, 0.02 substitutions per site. The tree is midpoint rooted for purposes of clarity only.

The Bayesian phylogenetic tree constructed with complete polyprotein sequences indicated that all Cuban isolates collected during the 2001-2002 epidemic grouped within genotype III, introduced in Latin America since 1994 (Fig. 1). Therefore, as expected, the Nicaraguan strain (NI_BID_V2420_1994) isolated around this time was located at the base of the Latin American group. All major nodes were statistically reliable according to the estimates of posterior probability. The phylogenetic tree further suggested that two lineages were circulating in Havana. It was noticeable that most Cuban isolates (20 isolates) representing the main lineage formed an independent monophyletic subgroup, closely related to Peruvian and Ecuadorian isolates from 2000 to 2002, but two isolates from the beginning of the epidemic(Cuba_118_2001 and Cuba_167_2001) appeared slightly distant, closely related to Venezuelan isolates from 2001.

### Genetic variability at the consensus sequence level.

Analysis of nucleotide sequences of the DENV-3 Cuban isolates, excluding samples Cuba_118_2001 and Cuba_167_2001, corresponding to a different lineage, revealed that relatively few nonsynonymous mutations occurred (Table 3). Notably, these mutations were generally unique for particular isolates, suggesting that they were not fixed within the population during the time of study, except for the mutation at nucleotide position 4380, which became fixed. This nonsynonymous mutation led to a conservative amino acid change of serine to asparagine, namely, S93N that is located in the NS2B protein. Puzzlingly, only the first isolate (Cuba_15_2001) had the S residue at this position, which was uncommon for DENV-3 strains of any genotype. Being a fairly indifferent amino acid, serine can reside both within the interior of a protein and on the protein surface. Its small size means that it is relatively common within tight turns on the protein surface, where it is possible for the serine side chain hydroxyl oxygen to form a hydrogen bond with the protein backbone, effectively mimicking proline. However, asparagine prefers generally to be on the surface of proteins, exposed to an aqueous environment (37).

**TABLE 3:** Amino acidic changes at the consensus level among the DENV-3 isolates collected at different time points during the Cuban epidemic, 2001 to 2002

Sample no.	Sample code	Amino acid residue of the viral protein at the indicated position:^*a*^																			
		C	E		NS1	NS2A		NS2B				NS3		NS4A	NS4B	NS5					
		39	198	329	256	4	195	33	59	60	93	60	155	39	18	190	246	250	371	637	830
1	Cuba_15_01	**K**	**T**	**V**	**H**	**K**	**A**	**V**	**I**	**T**	**S**	**H**	**V**	**R**	**E**	**I**	**H**	**T**	**R**	**H**	**V**
2	Cuba_26_01										N										
3	Cuba_73_01										N				D						
4	Cuba_118_01	N			Y	E	T		V		N	Y	I	K		V				R	I
5	Cuba_167_01	N			Y	E	T		V		N	Y	I	K		V				R	I
6	Cuba_463_01										N							A			
7	Cuba_492_01										N							A			
8	Cuba_504_01										N										
9	Cuba_513_01										N										
10	Cuba_523_01							L			N						Y				
11	Cuba_546_01										N										
12	Cuba_547_01			A							N										
13	Cuba_553_01										N										
14	Cuba_557_01			A							N										
15	Cuba_558_01										N										
16	Cuba_568_01										N								K		
17	Cuba_580_01										N										
18	Cuba_16_02									I	N										
19	Cuba_11_02										N										
20	Cuba_17_02		A								N										
21	Cuba_20_02										N										
22	Cuba_21_02										N										

a The reference sequence of Cuba_15_2001 is shown in boldface. Only substitutions are shown. Samples 4 and 5 correspond to a different lineage.

Finally, samples Cuba_553_2001 and Cuba_558_2001 collected 2 days apart from the same patient showed identical consensus nucleotide sequences.

### Viral population analysis.

**FIG 2:**
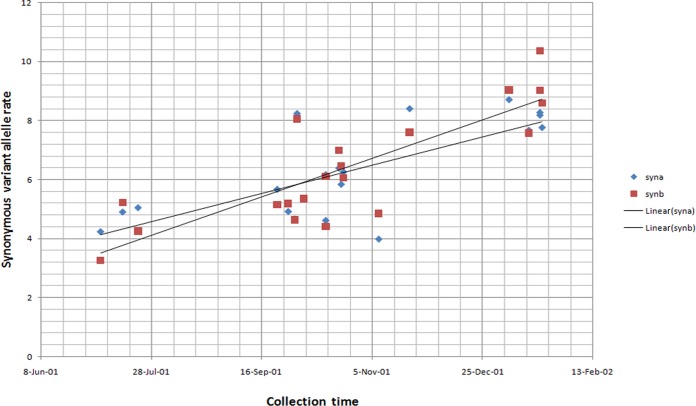
Synonymous variant allele rate per 10,000 bases at the complete-genome level according to time of sample collection during the 2001-2002 epidemic. Data sets a and b correspond to two different DNA libraries processed for each acute-phase sample through deep sequencing. For the synonymous variant allele rate in data set a (syna), *R*^2^ = 0.547, and for data set b (synb), *R*^2^ = 0,693; for the linear tendency for data set a, *P* = 1.94e−4, and for data set b, *P* = 5.19e−6.

Synonymous and nonsynonymous variant allele rates per 10,000 bases at the complete-genome level were calculated according to time of isolation. An increasing trend toward the end of the epidemic was observed only for synonymous variant allele rates (Fig. 2). Interestingly, in terms of nonsynonymous variant alleles, the viral population analysis indicated that at position 4380 in the NS2B gene, a significant minor population (A, 0.875%; T, 0.0%; C, 0.0%; G, 99.111%) present in the first isolate collected during the epidemic (Cuba_15_2001) was selected and became predominant (A, 99.943%; T, 0.0%; C, 0.0%; G, 0.664%) at the end of the epidemic. Taking into account this pattern, variants at low frequency (<1%) were considered relevant; therefore, unique significant minority variants (>0.1%) that appeared with the epidemic's progression were analyzed using the first isolate (Cuba_15_2001) as a reference.

**FIG 3:**
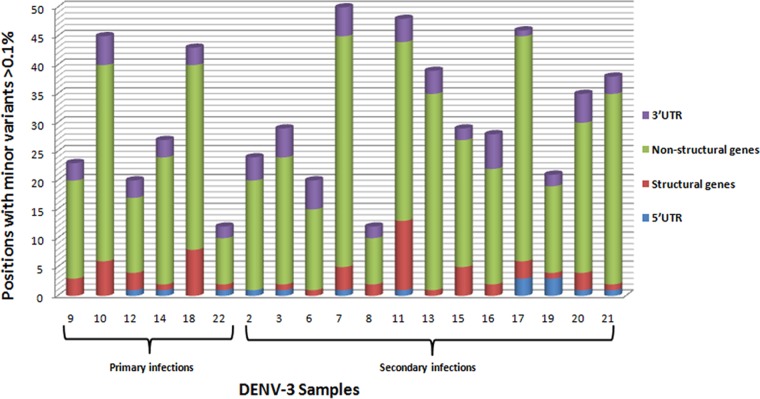
Number of positions with unique significant minority variants common for sets a and b (>0.1%), taking as a reference the first isolate of the DENV-3 Cuban epidemic of 2001 to 2002. Samples are grouped according to type of infection in primary and secondary infections by date of sample collection (Table 1). UTR, untranslated region.

Greater variability was observed in the nonstructural genes than in the structural genes in terms of significant minority variants, involving mainly NS5, NS3 and NS4B genes, and particularly toward the end of the epidemic. Across the genome, the number of positions with significant minority variants (>0.1%) ranged from 12 to as high as 48 in both primary and secondary infections without significant differences between these groups (Fig. 3).

**FIG 4:**
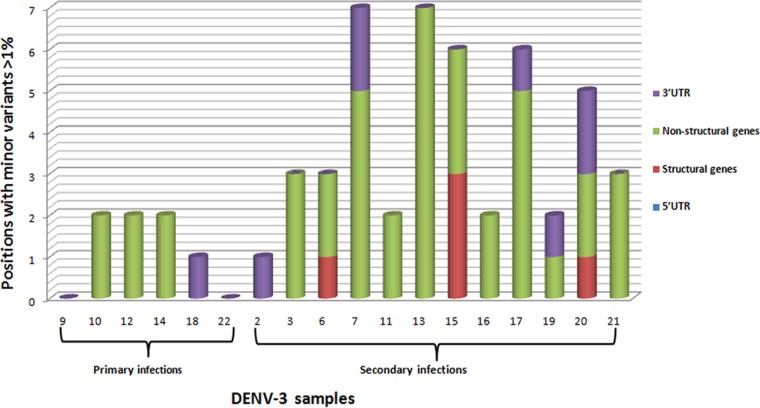
Number of positions with unique significant minority variants common for sets a and b (>1%), taking as a reference the first isolate of the DENV-3 Cuban epidemic of 2001 to 2002. Samples are grouped according to type of infection in primary and secondary infections by date of sample collection (Table 1).

However, different results were obtained when higher-frequency minority variants (>1%) were analyzed (Fig. 4). Still greater variability was observed in the nonstructural genes than in the structural genes, but it was noteworthy that patients with secondary infections showed greater variability than patients with primary infections. In addition, patients with secondary infections presented minority variants in the structural genes (PrM and E), some of which were nonsynonymous. In contrast, patients suffering primary infections had only mostly synonymous minority variants (>1%) in nonstructural genes (Tables 4 and 5).

**TABLE 4:** Unique significant variants (>1%) common for set a and b for primary infection cases taking as a reference the first isolate obtained during the Cuban epidemic, 2001 to 2002

Sample no.	Feature	Position (nt)^*c*^	Reference allele	Set a variant				Set b variant			
				Allele	Amino acid no.	Change	Frequency (%)	Allele	Amino acid no.	Change	Frequency (%)
9	−^*a*^	−	−	−	−	−	−	−	−	−	−
10	NS1	3424	C	T	344	Synonymous → N	1.145	T	344	Synonymous → N	1.037
	NS1	2887	C	T	165	Synonymous → T	1.079	T	165	Synonymous → T	0.966
12	NS5	9803	C	T	754	Synonymous → L	4.539	T	754	Synonymous → L	4.371
	NS5	7866	C	T	108	P → L	1.538	T	108	P → L	1.536
14	NS1	2924	C	T	178	Synonymous → L	5.312	T	178	Synonymous → L	6.398
	NS3	5371	C	T	293	Synonymous → A	1.086	T	293	Synonymous → A	0.134
18	3′ UTR	10369	G	T	*^b^	*	1.641	T	*	*	2.097
22	−	−	−	−	−	−	−	−	−	−	−

a Minority variants represent <1%.

b Indicates position in noncoding region of the 3′ untranslated region (UTR).

c nt, nucleotide.

**TABLE 5:** Unique significant variants (>1%) common for set a and b for secondary infection cases taking as a reference the first isolate obtained during the Cuban epidemic, 2001 to 2002^*a*^

Sample no.	Feature	Position (nt)^*b*^	Reference allele	Set a variant				Set b variant			
				Allele	Amino acid no.	Change	Frequency (%)	Allele	Amino acid no.	Change	Frequency (%)
2	3′ UTR	10404	G	A	*^c^	*	11.859	A	*	*	12.027
3	**NS4B**	**6853**	**G**	**C**	**18**	**E** → **D**	**64.984**	**C**	**18**	**E → D**	**62.998**
	NS5	8212	C	T	223	Synonymous → G	53.212	T	223	Synonymous → G	0.905
	2K peptide^*d*^	6751	C	T	7	Synonymous → L	26.347	T	7	Synonymous → L	26.578
	NS3	5956	C	T	488	Synonymous → H	24.414	T	488	Synonymous → H	24.625
6	PrM	519	C	T	35	T → I	5.444	T	35	T → I	4.605
	NS5	8080	T	C	179	Synonymous → I	3.055	C	179	Synonymous → I	1.961
	NS5	9743	C	T	734	Synonymous → L	2.825	T	734	Synonymous → L	1.11
7	**NS5**	**10000**	**G**	**A**	**819**	**Synonymous → E**	**52.534**	**A**	**819**	**Synonymous → E**	**52.01**
	3UTR	10481	C	T	*	*	27.601	T	*	*	27.616
	NS3	5098	G	A	202	Synonymous → R	6.53	A	202	Synonymous → R	7.952
	NS2A	3531	G	T	28	G → V	1.883	T	28	G → V	2.237
	3UTR	10359	C	T	*	*	1.881	T	*	*	1.805
	NS5	9265	C	T	574	Synonymous → N	1.339	T	574	Synonymous → N	1.423
	NS4B	7366	C	A	189	Synonymous → A	1.093	A	189	Synonymous → A	1.417
8	NS2B	4294	G	A	64	Synonymous → E	7.278	A	64	Synonymous → E	7.562
11	**E**	**1834**	**G**	**A**	**307**	**Synonymous → K**	**99.597**	**A**	**307**	**Synonymous → K**	**99.93**
	**E**	**991**	**G**	**A**	**26**	**Synonymous → E**	**99.584**	**A**	**26**	**Synonymous → E**	**99.862**
	NS5	10049	G	A	836	V → I	7.903	A	836	V → I	7.418
	NS5	10223	G	C	894	E → Q	6.094	C	894	E → Q	6.061
13	**NS5**	**9142**	**C**	**T**	**533**	**Synonymous → D**	**99.288**	**T**	**533**	**Synonymous → D**	**98.715**
	**NS3**	**5371**	**C**	**T**	**293**	**Synonymous → A**	**96.832**	**T**	**293**	**Synonymous → A**	**99.878**
	NS5	9985	C	T	814	Synonymous → N	3.203	T	814	Synonymous → N	3.15
	NS4B	7012	G	A	71	Synonymous → Q	1.723	A	71	Synonymous → Q	1.678
	NS5	7975	G	A	144	Synonymous → L	1.511	A	144	Synonymous → L	1.686
	NS5	7744	G	A	67	Synonymous → E	1.472	A	67	Synonymous → E	1.484
	NS3	5326	C	T	278	Synonymous → N	1.386	T	278	Synonymous → N	1.003
	NS4B	7366	C	T	189	Synonymous → A	1.259	T	189	Synonymous → A	0.921
	NS2A	3500	C	T	18	L → F	0.983	T	18	L → F	1.474
15	**NS5**	**9142**	**C**	**T**	**533**	**Synonymous → D**	**98.963**	**T**	**533**	**Synonymous → D**	**97.096**
	**NS3**	**5371**	**C**	**T**	**293**	**Synonymous → A**	**97.515**	**T**	**293**	**Synonymous → A**	**99.666**
	NS2B	4315	C	T	71	Synonymous → S	3.495	T	71	Synonymous → S	5.037
	E	1510	G	A	199	M → I	2.366	A	199	M → I	2.212
	NS5	9092	A	G	517	I → V	1.579	G	517	I → V	1.941
	PrM	442	G	T	9	E → D	1.114	T	9	E → D	1.283
	NS1	3373	C	T	327	Synonymous → D	1.096	T	327	Synonymous → D	1.032
	E	2235	C	T	441	A → V	0.914	T	441	A → V	0.902
16	**NS5**	**8655**	**G**	**A**	**371**	**R** → **K**	**99.491**	**A**	**371**	**R** → **K**	**98.923**
	NS3	6184	C	T	564	Synonymous → C	6.696	T	564	Synonymous → C	6.419
	NS4B	7444	C	A	215	Synonymous → T	1.848	A	215	Synonymous → T	2.41
17	**NS1**	**3310**	**G**	**A**	**306**	**Synonymous → K**	**99.055**	**A**	**306**	**Synonymous → K**	**99.847**
	NS5	8428	C	T	295	Synonymous → D	13.026	T	295	Synonymous → D	96.876
	NS5	8875	C	T	444	Synonymous → G	2.936	T	444	Synonymous → G	0.075
	NS5	9127	C	T	528	Synonymous → A	1.875	T	528	Synonymous → A	0.102
	3UTR	10283	C	T	*	*	1.817	T	*	*	0.067
	NS5	8659	C	T	372	Synonymous → V	1.143	T	372	Synonymous → V	0.086
	NS5	8743	G	A	400	Synonymous → K	0.894	A	400	Synonymous → K	0.09
	NS5	7594	A	G	17	Synonymous → L	0.856	G	17	Synonymous → L	1.16
19	NS4A	6574	T	C	75	Synonymous → G	1.48	C	75	Synonymous → G	1.116
	3UTR	10661	A	G			0.6	G			0.838
20	**NS3**	**5791**	**G**	**A**	**433**	**Synonymous → V**	**96.402**	**A**	**433**	**Synonymous → V**	**99.509**
	PrM	652	G	T	79	Synonymous → T	4.65	A	79	Synonymous → T	0.073
	3UTR	10514	G	C	*	*	3.956	C	*	*	4.416
	NS3	4685	C	T	65	Synonymous → L	3.66	T	65	Synonymous → L	0.286
	3UTR	10577	A	C	*	*	2.459	C	*	*	2.533
	2K peptide	6769	C	A	13	Synonymous → G	2.048	A	13	Synonymous → G	2.463
21	NS5	8212	C	T	223	Synonymous → G	39.457	T	223	Synonymous → G	97.343
	NS4A	6700	G	T	117	Synonymous → V	8.487	T	117	Synonymous → V	8.528
	NS4B	6853	G	C	18	E → D	1.086	C	18	E → D	0.797

a Boldface indicate that these variants became predominant in the particular patient.

b nt, nucleotide.

c Indicates positions in noncoding region of the 3′ untranslated region (UTR).

d 2K peptide, the DENV 2K-signal sequence is a 17-amino-acid peptide linking NS4A with NS4B.

**FIG 5:**
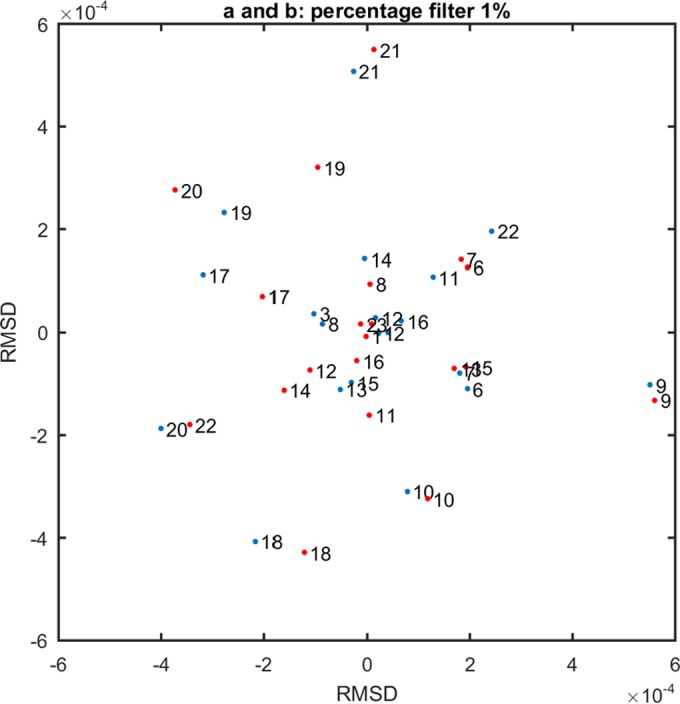
Multidimensional scaling using root mean square deviation (RMSD) values calculated using significant minority variants (>1%) for data set a (red dots) and b (blue dots). Numbers represent the 20 studied samples ordered by collection time, as indicated in Table 1. Samples 4 and 5 that correspond to a different lineage were excluded.

**FIG 6:**
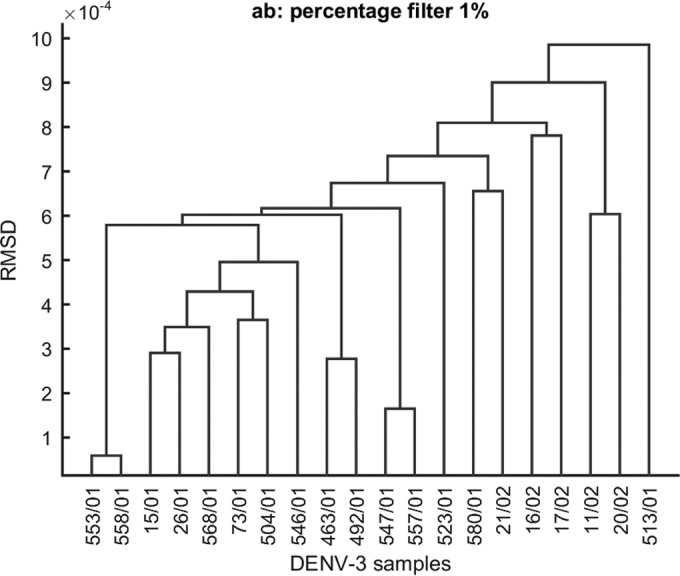
Dendrogram clustering of Cuban isolates collected at different time points during the 2001-2002 epidemic using an RMSD-based distance matrix including data sets a and b. Samples that correspond to a different lineage were excluded.

Intersample cluster analyses using RMSD values based on MDS showed greater variability with the epidemic's progression. Samples with similar characteristics were reflected in the plot by their close spatial proximity to each other. Isolates collected at the end of the epidemic were located on the periphery of the plotting area, indicating higher variability (Fig. 5). Dendrograms using unique significant minority variants (>0.1%, >0.5%, and >1%) showed similar results; isolates collected at the very beginning were closely related and had less genetic variability than late isolates, based on RMSD values (Fig. 6).

Finally, significant minority variants present in samples Cuba_553_2001 and Cuba_558_2001 collected from the same patient at days 2 and 4 after fever onset were compared (Table 6). The analysis revealed changes in the viral population structure during the course of a secondary infection that were well supported by our data since high-quality sequences were obtained (Table 6), and similar results were obtained in two independent DNA libraries (a and b). Two silent nucleotide substitutions, C/T at position 5371 in NS3 and C/T at position 9142 in NS5, were found in both samples as the predominant population. Notably, these variants were present as significant minority variants in the first isolate, Cuba_15_2001. In addition, seven unique significant minority variants (>1%) were found in sample Cuba_553_2001 that were absent in sample Cuba_558_2001 (six of which were synonymous). Likewise, six unique significant minority variants (>1%) were found in sample Cuba_558_2001 that were absent in sample Cuba_553_2001 (four of which were nonsynonymous). Interestingly, significant minority variants in the sample collected at day 2 corresponded exclusively to nonstructural genes, while significant minority variants present in the sample collected at day 4 included nonsynonymous changes in the PrM, E, and NS5 genes. The change E9D in PrM protein was located in the N terminus of Pr near motif 6, one of the prominent complementary electrostatic patches in the PrM-E heterodimer. The changes M199I and A441V in E protein were located in domain II and the membrane-proximal stem (EH2), respectively. The stem has two predicted amphipathic helices that lie half buried in the outer leaflet of the viral membrane. For fusion to take place, the stem region must span the length of the domain II region. In addition, EH2 can affect the expression and stability of its chaperone PrM (38–40). Finally, the change I517V in NS5 protein was located in the RNA-dependent RNA polymerase catalytic domain (41). Simultaneous with the intrahost genetic diversity observed during the course of infection in this particular patient, increasing neutralizing titers were observed for DENV-1 (from 1/28 to 1/200) as well for DENV-3 (from <1/10 to 1/200) in samples collected at days 2 and 4 after fever onset. Nevertheless, titers for DENV-2 and DENV-4 were undetectable (<1/10) at days 2 and 4 after fever onset. At day 5, neutralizing titers were considerably elevated against all DENV serotypes.

**TABLE 6:** Unique significant variants (>1%) common for set a and b present in two samples corresponding to the same patient at days 2 and 4 after fever onset, taking as a reference the first isolate obtained during the Cuban epidemic, 2001 to 2002^*a*^

Sample and feature	Position (nt)^*b*^	Read coverage^*d*^	Reference allele	Set a variant				Set b variant			
				Allele	Amino acid no.	Change	Frequency (%)	Allele	Amino acid no.	Change	Frequency (%)
Cuba_553_2001											
**NS5**	**9142**	**30,246**	**C**	**T**	**533**	**Synonymous → D**	**99.288**	**T**	**533**	**Synonymous → D**	**98.715**
**NS3**	**5371**	**8,024**	**C**	**T**	**293**	**Synonymous → A**	**96.832**	**T**	**293**	**Synonymous → A**	**99.878**
NS5	9985	30,249	C	T	814	Synonymous → N	3.203	T	814	Synonymous → N	3.15
NS4B	7012	16,819	G	A	71	Synonymous → Q	1.723	A	71	Synonymous → Q	1.678
NS5	7975	19,551	G	A	144	Synonymous → L	1.511	A	144	Synonymous → L	1.686
NS5	7744	18,047	G	A	67	Synonymous → E	1.472	A	67	Synonymous → E	1.484
NS3	5326	8,654	C	T	278	Synonymous → N	1.386	T	278	Synonymous → N	1.003
NS4B	7366	15,691	C	T	189	Synonymous → A	1.259	T	189	Synonymous → A	0.921
NS2A	3500	12,713	C	T	18	L → F	0.983	T	18	L → F	1.474
Cuba_558_2001											
**NS5**	**9142**	**13,986**	**C**	**T**	**533**	**Synonymous → D**	**98.963**	**T**	**533**	**Synonymous → D**	**97.096**
**NS3**	**5371**	**18,626**	**C**	**T**	**293**	**Synonymous → A**	**97.515**	**T**	**293**	**Synonymous → A**	**99.666**
NS2B	4315	27,529	C	T	71	Synonymous → S	3.495	T	71	Synonymous → S	5.037
E	1510	24,416	G	A	199	M → I	2.366	A	199	M → I	2.212
NS5	9092	12,493	A	G	517	I → V	1.579	G	517	I → V	1.941
PrM	442^*c*^	19,513	G	T	9	E → D	1.114	T	9	E → D	1.283
NS1	3373	18,492	C	T	327	synonymous>D	1.096	T	327	Synonymous → D	1.032
E	2235	30,874	C	T	441	A → V	0.914	T	441	A → V	0.902

a Boldface indicate that this variant became predominant.

b nt, nucleotide.

c Variant A (synonymous → E) at position PrM 442 was detected in sample Cuba_553_2001 at a frequency of 0.1% (day 2).

d Read coverage, the average number of reads that align to each base of the reference sequence.

## DISCUSSION

Most epidemiological inferences made to date from genomic sequence analysis of RNA viruses, particularly of dengue virus, have used the consensus sequences of viral populations. However, it is becoming progressively clearer that increases in fitness and changes in adaptability are observed without changes in the consensus sequence (42). Next-generation sequencing is greatly expanding the capacity to examine the composition of mutant spectra in infected cells and host organisms with unprecedented accuracy. Therefore, significant progress in the understanding of viral population dynamics has been made in recent years (43, 44).

In dengue virus, the association between intrahost diversity and disease outcome is an area of active investigation (7). Unfortunately, the comparison among available publications is complex and inadequate, taking into account that different genes are examined by dissimilar approaches and that dissimilar epidemiological settings are evaluated in terms of DENV circulating serotypes/genotypes and the immunological background of the population.

Based on the evidence that dengue virus fitness is context dependent, better characterization of viral evolution in exceptional epidemiological settings could contribute to a better understanding of dengue pathogenesis. However, few studies have been able to properly demonstrate that the dynamics of dengue disease severity is determined by the interplay between viral genetics and serotype-specific immunity (10, 14, 45) because dengue is hyperendemic in most tropical countries. The present study is unique because it is the first that characterizes the entire viral population of DENV-3 from samples collected at different time points during a single epidemic taking place in a well-characterized epidemiological context with only one serotype circulating following a 20-year period of total absence of dengue, with a demonstrated intraepidemic increase in clinical severity.

Phylogenetic analyses based on a full-length consensus sequence were consistent with those previously obtained using E gene sequences, showing that the etiological agent of the epidemic belongs to genotype III (46). However, the higher number of samples studied at present revealed that two introductions occurred during this epidemic period. Presumably, according to the data set, the transmission of the minor lineage represented by two isolates was short-lived. While we cannot exclude an undersampling effect, this minor lineage was detected only in the Arroyo Naranjo municipality at the beginning of the epidemic. Afterwards, only isolates genetically related to the main lineage were collected at this location. Clearly, the minor lineage could have been eliminated due to vector control actions in a particular area of Arroyo Naranjo close to the time of introduction. Alternatively, the minor lineage could have been out-competed due to a lower viral fitness than that of the main lineage. Previous studies on the biological properties of DENV-3 isolated during this epidemic indicated that strains corresponding to the less representative lineage (Cuba_118_2001 and Cuba_167_200) collected in Arroyo Naranjo were more sensitive to high temperature (39°C) during replication in BHK-21 cells than strains corresponding to the main lineage (47). In addition, a study on the calculation of the basic reproductive number, *R*_0_ in different municipalities of Havana indicated that Arroyo Naranjo municipality had the lowest *R*_0_ estimated value, i.e., 1.97 (95% confidence interval [CI], 1.94 to 2.01) whereas Boyeros (48), one of the last municipalities affected by the epidemic, had an *R*_0_ of 61.06 (95% CI, 60.44 to 61.68).

Considering the main lineage as the one involved in the phenomenon of increasing clinical severity, temporary changes that appear at the consensus level in the 20 DENV-3 samples conforming to this lineage were analyzed. Contrary to expectations, the clear pattern of evolution observed during the 1997 Cuban epidemic (14) was not demonstrated here. A unique amino acid change, S93N in NS2B, differentiates all the studied isolates from the first isolate of the epidemic. Interestingly, only the first isolate had serine at this position, which is uncommon for DENV of any serotype. However, according to deep sequencing, the genetic variant coding for the common motif asparagine was also present in the first isolate as a minor variant (<1%).The fact that this motif remains invariant for DENV-3 at deeper phylogenetic levels suggests that it is favored in nature. Importantly, the replication of DENV requires the correct processing of the polyprotein by the viral NS3 protease (NS3pro). For full enzymatic activity NS3pro requires the hydrophilic part of the integral membrane protein NS2B as a cofactor (residues 49 to 95), which includes the position of change (S93N) (49–51).

In contrast to the low variation observed at the consensus level with the epidemic's progression, the viral population analysis from patients infected at different time points during the epidemic revealed greater variability in terms of significant minority variants in the nonstructural genes than in structural genes, particularly toward the end of the epidemic. However, it is remarkable that the increasing trend toward the end of the epidemic was observed only for synonymous mutations, except for the mutation in NS2B. Nonetheless, its implication for viral fitness should not be underestimated. Previous studies on vesicular stomatitis virus indicate that a significant fraction of silent mutations are not neutral and can significantly contribute to adaptation since a large part of this sequence space is under natural selection, with an impact on viral fitness (52). Likewise, a recent study that accurately describes poliovirus populations demonstrated that a significant fraction of synonymous changes are subject to strong selection (44). Indeed, there is evidence that many synonymous sites in RNA viruses are not neutral and clearly impact viral fitness (53, 54).

According to Domingo et al., the advantage of a broad mutant spectrum lies in the increased capacity of the viral population to find portions of sequence space in which to increase its fitness (55). In the present study, viral population analysis also showed greater variability in patients with secondary infections, including significant minority variants (>1%) that imply changes in the structural genes not present in patients with primary infections. One of the most striking observations from this study was the intrahost genetic variation observed within the same patient during the course of infection. The viral population changed in 2 days; remarkably, new variants that arose on day 4 presented nonsynonymous changes at the structural genes PrM and E. Moreover, changes in the antibody response profile were demonstrated during the acute phase in this patient.

Could this variability depend on the immunity of the patient? According to previous seroepidemiological studies, all severe cases observed during the 2001-2002 Cuban epidemic had the sequence of DENV-1 infection followed by DENV-3 (DENV-1/DENV-3) or experienced tertiary infection (DENV-1/DENV-2/DENV-3) (56). However, the infection sequence DENV-2/DENV-3 was associated with asymptomatic infection or mild disease (57). Similar results were observed in a different epidemiological setting (58), and it is suggested that antibodies against DENV-2 could have the ability to neutralize and downregulate DENV-3 infections. In accordance, a recent study that characterized the antigenic diversity in the DENV types by antigenic maps constructed from neutralizing antibody titers has shown that whereas DENV isolates are usually located closer to other viruses of the same type, some viruses, both modern and historical, have greater antigenic resemblance to viruses of a different type than to some viruses of the same type (59). Therefore, the viral population transmitted to a healthy individual from an infected mosquito may vary depending on the immunological background of the previous individual on which the mosquito fed. Likewise, the immunological background of the new human host could have an effect on the viral population during the course of infection. Recently, Sim et al. used whole-genome amplification and next-generation sequencing to characterize DENV intrahost genetic diversity in both patient-derived and matched-mosquito-derived virus populations. Mosquitoes were infected by direct feeding on patients, enabling the authors to track changes in viral populations during human-to-mosquito transmission. However, changes in viral populations during transmission from mosquito back to human were not examined, and the immunological background of the patients enrolled in the study was not determined (20).

In the present situation, the DENV-3 mutant spectrum could be replicating in total absence of DENV heterologous antibodies during many cycles of primary infections since a large part of the population was naive (at least all individuals born after 1981). Alternatively, during secondary infections in the sequence DENV-1/DENV-3, higher viral load could be expected via antibody-dependent enhancement. Viral load, together with population genetic heterogeneity, permits exploration of sequence space for a fitness increase. As viral fitness is environment and population size dependent, it can change according to the immunological landscape.

The emergence of significant minority variants with changes in the structural proteins PrM and E late during the acute phase of the disease in a secondary infection case (DENV-1/DENV-3) is described for the first time in dengue. However, its implications concerning pathogenesis should be examined with a higher number of well-characterized serial samples, with data including the immunological background of the patients. The high-resolution analysis of intrahost genetic diversity published by Thai et al. in serial plasma samples taken from 17 patients infected with DENV-1 revealed that nucleotide sequence diversities of viral populations were very low, ranging from 0 to 0.0013 among different samples collected from the same patient (17). While this study explored only the E gene (exclusively the fragment coding domain III) and used cloning techniques rather than deep sequencing, its observations fit within the results of the present study, which observed the lowest diversity in the structural genes. Moreover, the significant minority variants with changes in the E gene that emerged in the same patient late during the course of infection did not involve domain III. Lack of diversity in the E gene has also been attributed to strong purifying selection (60).

In conclusion, our results suggest that changes in the viral population swarm occurred with the epidemic's progression and that these changes could have had an impact on viral fitness. Therefore, the dynamics of evolving viral populations in the context of heterotypic antibodies could be related to the increasing clinical severity observed during dengue epidemics. Definitely, new experiments are required to prove *in vivo* and/or *ex vivo* the role of particular mutations on the increased viral fitness toward the end of the epidemic. In this regard, the impact of synonymous alleles deserves further study, focused on the biological basis of the selective advantage of silent mutations in DENVs. More importantly, studies addressing the extent and pattern of intrahost genetic diversity during the course of dengue secondary infections using deep-sequencing approaches should be tackled to unravel molecular mechanisms involved in dengue virus pathogenesis.
